# Production of Therapeutically Relevant Indolizidine Alkaloids in *Securinega suffruticosa* In Vitro Shoots Maintained in Liquid Culture Systems

**DOI:** 10.1007/s12010-014-1386-0

**Published:** 2014-11-21

**Authors:** Danuta Raj, Adam Kokotkiewicz, Maria Luczkiewicz

**Affiliations:** 1 The Chair and Department of Pharmacognosy, Faculty of Pharmacy, Wroclaw Medical University, ul. Borowska 211 A, 50-556 Wrocław, Poland; 2 The Chair and Department of Pharmacognosy, Faculty of Pharmacy, Medical University of Gdansk, al. gen. J. Hallera 107, 80-416 Gdańsk, Poland

**Keywords:** Allosecurinine, Auxins, Bubble column bioreactor, Cytokinins, Indolizidine alkaloids, Microshoots, Phyllanthaceae, Securinine

## Abstract

Microshoot cultures of the Chinese medicinal plant *Securinega suffruticosa* (Pall.) Rehd. were established and evaluated for the presence of therapeutically relevant indolizidine alkaloids securinine (S) and allosecurinine (AS). The cultures were maintained in shake flasks (SFs) and a bubble column bioreactor (BCB) using the modified Murashige’s shoot multiplication medium supplemented with 1.0 mg l^−1^ benzyladenine (BA), 3.0 mg l^−1^ 2-isopentenyladenine (2iP), and 0.3 mg l^−1^ 1-naphthaleneacetic acid (NAA). The influence of light and medium supplementation strategies with biosynthesis precursor (lysine (LY)) and nutrient formulations (casein hydrolysate (CH) and coconut water (CW)) on biomass growth and alkaloid production were investigated. SF cultures grown in the presence of light yielded up to 6.02 mg g^−1^ dry weight (DW) S and 3.70 mg g^−1^ DW AS, corresponding to the respective productivities of 98.39 and 60.21 mg l^−1^. Among feeding experiments, CW supplementation proved most effective for SF-grown shoots, increasing biomass yield and AS productivity by 52 and 44 %, respectively. Maximum concentrations of securinine (3.25 mg g^−1^ DW) and allosecurinine (3.41 mg g^−1^ DW) in BCB cultures were achieved in the case of 1.0 g l^−1^ LY supplementation. These values corresponded to the productivities of 42.64 and 44.47 mg per bioreactor, respectively.

## Introduction

*Securinega suffruticosa* (Pall.) Rehd. (Phyllanthaceae) is a shrub of Asiatic origin, mostly found in Manchuria and internal Mongolia. In Traditional Chinese Medicine, *S. suffruticosa* constitutes one of the 50 basic medicinal plants and is used for the treatment of impotence, paralysis, quadriplegia, rheumatic diseases, and mental disorders [1]. The plant owes its therapeutic potential to the presence of indolizidine alkaloids, represented mainly by securinine. As an antagonist of *γ*-aminobutyric acid A (GABA_A_) receptor and cholinergic agent, securinine was shown to exhibit beneficial effects in a number of neurological disorders. The drug may be useful for the therapy of memory impairment and neurodegenerative diseases such as Alzheimer’s [2, 3]. Remarkable results were also obtained in people suffering from amyotrophic lateral sclerosis, where securinine significantly improved life quality [2]. Apart from the above activities, the alkaloid was demonstrated to possess anticancer properties: It induced human colon cancer cell autophagy and apoptosis [4, 5], decreased metastasis in colorectal cancer patients [6], and has potential as an acute myeloid leukaemia therapeutic [7]. Securinine was also shown to stimulate macrophage activation and thus may be useful in the management of infections. In the study by Holmes and co-workers [8], the alkaloid inhibited toxoplasma growth.

Given the high therapeutic potential of securinine, establishing an efficient method for its production may be of interest. However, the chemical synthesis of the discussed compound is rather complicated [9] whereas traditional farming is vulnerable to climate changes, pest, and herbivore attack [10].

In the presented work, in vitro shoot cultures of *S. suffruticosa* were evaluated as an alternative, renewable source of indolizidine alkaloids. Liquid cultures of the discussed plant were obtained and subjected to different experimental strategies aimed at increasing secondary metabolite yield, including the modifications of the light regime (photoperiod or complete darkness) and medium supplementation with biosynthesis precursors (lysine hydrochloride (LY)) and nutrient formulations (casein hydrolysate (CH) and coconut water (CW)) [10]. The in vitro shoots were subsequently adapted to the growth in a laboratory-scale column bioreactor. The established cultures were evaluated for indolizidine alkaloid content and productivity.

## Materials and Methods

### In Vitro Culture Reagents, Procedures, and Conditions

The reagents used for media preparation and feeding experiments were from Sigma-Aldrich (Sigma-Aldrich, St. Louis, MO, USA). Water was produced using REL 5 double water still (POLNA, Przemysl, Poland). All culture media were supplemented with 3.0 % *w*/*v* sucrose, and stationary cultures were solidified with 0.7 % *w*/*v* agar. The pH was adjusted to 5.8 prior to autoclaving (0.1 MPa, 121 °C, 21 min). Thermolabile plant growth regulators (PGRs; indole-3-acetic acid (IAA) and 2-isopentenyladenine (2iP)) were added post-autoclaving via sterile filtration (0.2 μm filters, FP 3070, 2 CA-S, Schleicher and Schuell, Keene, NH, USA). Solutions of CH and LY were sterile filtered into the media whereas the sterile manufactured CW was directly poured into the growth vessels under aseptic conditions.

Unless otherwise stated, the cultures were maintained in a growth chamber at 24 ± 1 °C, under a 16 h (light)/8 h (dark) photoperiod (white fluorescent lamps, 36 W, light intensity 88 ± 8 μmol m^−2^ s^−1^, Philips, Amsterdam, The Netherlands). The experiments were conducted in two series, and the results presented as mean of two samples ± SD.

### Plant Material

**Fig. 1:**
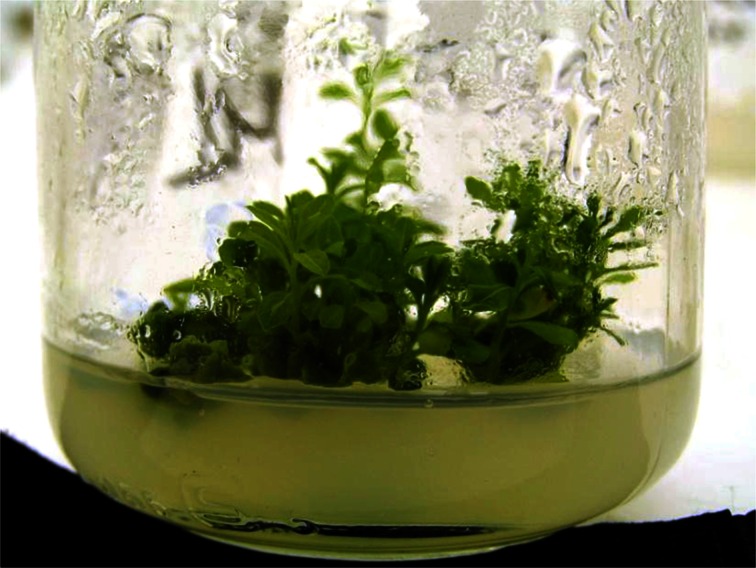
*S. suffruticosa* microshoots grown on stationary “Murashige’s shoot multiplication ‘A’ medium” supplemented with 0.3 mg l^−1^ NAA, 3.0 mg l^−1^ 2iP, and 1.0 mg l^−1^ BA

Microshoot cultures were established from *S. suffruticosa* seeds, acquired from Wroclaw University Botanical Garden and deposited at number 003/WP at the herbarium of the Department of Pharmacognosy, Faculty of Pharmacy, Wroclaw Medical University. Before germination, the seeds were pre-washed with 1 % commercial detergent for 10 min, followed by 30 s treatment with 70 % *v*/*v* aqueous ethanol. The main sterilization was conducted with sodium hypochlorite (10 % solution of commercial bleach “Domestos”, Unilever Polska, Warszawa, Poland) for 30 min. The seeds were rinsed three times with sterile, double distilled water, placed into petri dishes lined with wet filtration paper, and held in the dark at 24 ± 1 °C. As the seeds germinated, the dishes were moved to a growth chamber for 2 weeks. The obtained seedlings were strengthened for 14 daya on the Murashige and Skoog (MS) medium [11] without PGRs. Further stages of shoot culture initiation involved the use of rich growth media (variants of MS and Schenk and Hildebrandt (SH) media), which were successfully applied during initial studies on in vitro cultures of *Securinega* [12]. The cotyledone fragments of the seedlings were excised and placed onto SH medium [13] enriched with 2.0 mg l^−1^ IAA and 0.5 mg l^−1^ 2,4-dichlorophenoxyacetic acid (2,4-D). After three subsequent 28-day passages, the formed initial callus was transferred onto “Murashige’s shoot multiplication ‘A’ medium” [14] with modified PGR composition (0.3 mg l^−1^ 1-naphthaleneacetic acid (NAA), 3.0 mg l^−1^ 2iP, and 1.0 mg l^−1^ benzyladenine (BA)), hereinafter referred to as ‘M_A_’ medium, in order to initiate shoot formation. The explants had been subcultured every 4 weeks until they formed a stable microshoot culture (Fig. 1). For the experiments, the portions of shoot biomass were taken on 28 days of the growth cycle. In the case of experiments run without the presence of light, the shoots were kept darkened for 28 days prior to use.

### Cultures Grown in Erlenmeyer Flasks

In order to initiate liquid shoot cultures, 3.0 g of the inoculum were inserted into 250 ml Erlenmeyer flasks containing 100 ml liquid M_A_ medium and plugged with silicone foam stoppers (Carl Roth, Karlsruhe, Germany). The cultures were maintained on rotary shaker (120 rpm, 25.4 mm orbit, INNOVA 2300, Eppendorf, Enfield, US-CT) under photoperiod or without the presence of light. Biomass and medium samples were harvested at 2-day intervals until 52 days of experiment.

During the feeding experiments, the cultures were supplemented with LY (1.0 g l^−1^), CH (1.0 g l^−1^), CW (10 % *v*/*v*), or their mixture (MIX, 1.0 + 1.0 g l^−1^ + 10 % *v/v*, respectively) on 34 days of the growth period and harvested on 44 days.

### Bioreactor Experiments

The details of bubble column bioreactor (BCB) employed for the current study were the same as in the previous work [15], except that the immobilization basket was placed 50 mm above the bottom of the growth vessel. For the experiment, the bioreactor was inoculated with 15.0 g of microshoots and filled with 600 ml of M_A_ medium. Aeration rate was kept at a constant 800 ml min^−1^ during the whole growth period. The experiment was run in a fed-batch mode, with 200 ml portions of fresh M_A_ medium added on 20 and 40 days. Biomass and medium samples were harvested at 10-day intervals until 70 days of the experiment.

During the feeding experiments, the cultures were supplemented with LY (1.0 g l^−1^), added on 0 or 20 days of the experiment, and harvested on 40 days.

### Evaluation of Indolizidine Alkaloid Content

The harvested biomass and media samples were stored at −18 °C prior to analysis. The shoots were freeze-dried (Lyovac GT2, Finn-Aqua-Santasalo-Sohlberg, Finland), homogenized, and subjected to a classical acidic-alkaline extraction mode. After being brought to room temperature, the respective media samples followed the same extraction procedure. Quantitative analysis of securinine and allosecurinine was performed by HPTLC with densitometric detection. The detailed description of the method was reported previously [16].

### Statistical Analysis

The obtained results were statistically evaluated with the Kruskal-Wallis analysis of variance by ranks, using Statistica 10.0 software (StatSoft Inc., USA).

## Results and Discussion

### The Effect of Light Regime on Biomass Yield and Alkaloid Content in Shake Flask Grown Microshoot Cultures

**Fig. 2:**
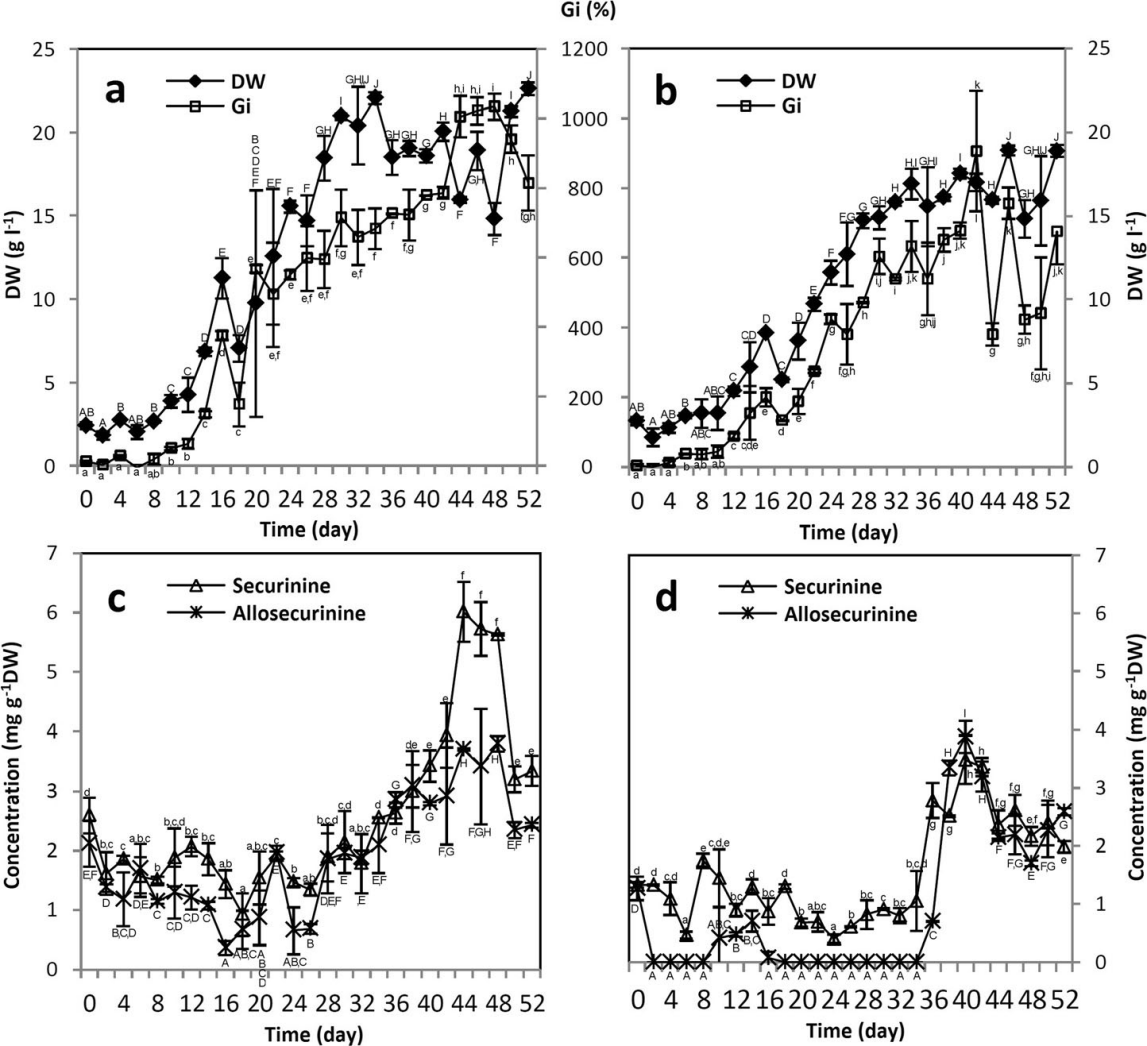
Changes in dry weight (DW), growth index (Gi), and indolizidine alkaloid concentrations in shake flask-grown *S. suffruticosa* shoot cultures maintained under photoperiod (**a**, **c**) and without the presence of light (**b**, **d**). Values represent the mean ± SD of two replicates. For each parameter, distinct signs indicate significant differences among the means (*p* ≤ 0.05)

**Fig. 3:**
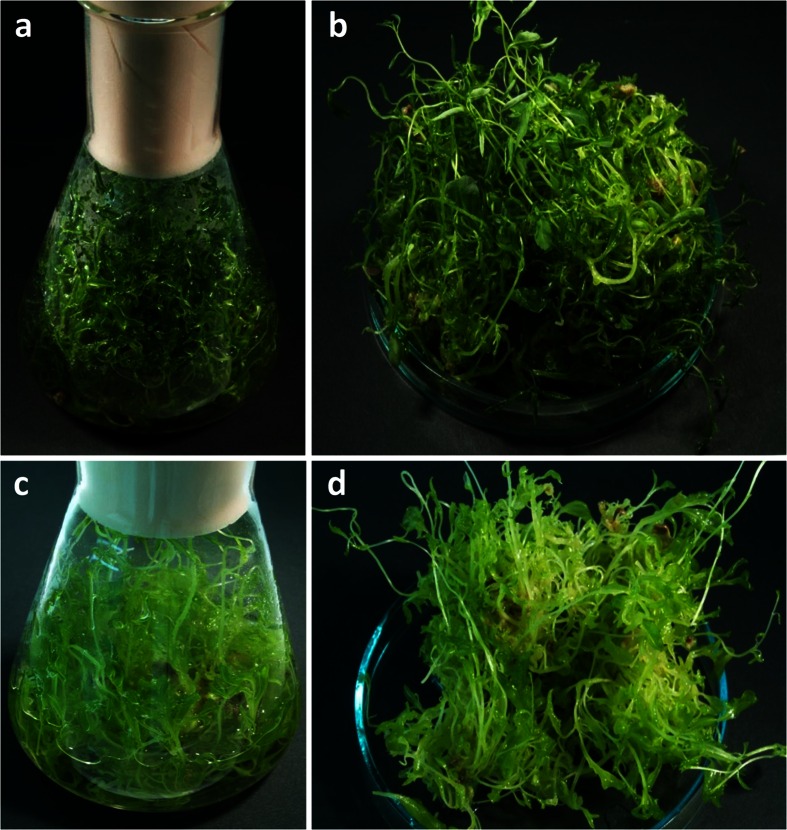
Shake flask-grown *S. suffruticosa* shoot cultures maintained for 52 days under photoperiod (**a**, **b**) and without the presence of light (**c**, **d**)

As shown in Fig. 2a, the culture maintained under photoperiod had relatively a long growth cycle with noticeable lag phase (L) from 0 to 10 days, logarithmic growth phase (G) from 12 to 32 days, and stationary phase (St) from 34 to 52 days. *S. suffruticosa* microshoots well tolerated liquid medium conditions, showing no signs of necrosis and completely filling the growth vessel during the 52-day culture period (Fig. 3a, b). Maximum Gi values exceeded 1000 % (Fig. 2a), indicating that the examined culture is prospective for large-scale cultivation.

The patterns of indolizidine alkaloid accumulation in photoperiod-grown microshoots are depicted in Fig. 2c. Phytochemical analysis revealed that S and AS were stored mostly intracellularly, with only traces (≤0.5 mg l^−1^) detected in the growth media. Therefore, extracellular alkaloids were omitted in further discussion.

Up to the 24 days of the experiment, there was a marked decrease in S and AS concentrations, corresponding with the phases of adaptation and intensive cell growth (Fig. 2a, c). The S/AS ratio slightly exceeded 1 for the first two thirds of the growth period and increased to nearly 2 during the stationary phase. Peak concentrations of the alkaloids (6.02 and 3.70 mg g^−1^ dry weight (DW) for S and AS, respectively) were recorded on 44 days (Fig. 2c), while maximal productivity per day (mg l^−1^ day^−1^) was set on 46 days (Table 1). It is noteworthy that S content in the investigated microshoot culture was ca. four times higher than in the intact plant material (1.5 mg g^−1^ DW [16]) and ca. 15–20 times higher than maximum S concentrations recorded in *S. suffruticosa* callus (0.3–0.4 mg g^−1^ DW [16, 17]). Statistical analysis showed significant differences (*p* ≤ 0.05) between L, G, and St phases both in terms of growth parameters (Gi, DW) and alkaloid accumulation.

**Table 1:** Maximal productivities per day of securinine (S) and allosecurinine (AS) in selected *S. suffruticosa* microshoot cultures grown under different experimental conditions

Experimental conditions	Day of the culture	Productivity (mg l^−1^ day^−1^)	
		S	AS
Shake flasks (photoperiod)	46	2.35	1.55
Shake flasks (darkness)	40	1.53	1.70
Shake flasks (+CW)	44	2.08	2.11
BCB	40	0.71	1.03
BCB LY d20	40	1.39	1.33

CW, coconut water (10 % *v*/*v*), added on the 34 days; BCB, bubble column bioreactor; LY d20, lysine (1.0 g l^−1^), added on the 20 days

Further part of the study was aimed at establishing the effect of the absence of light on the growth and alkaloid content of *S. suffruticosa* microshoots. Dark cultivation of plant cells offers some benefits regarding large-scale systems, such as reduced process costs and lack of problems associated with homogeneous irradiation of bioreactor-grown biomass. Moreover, in the previous experiments concerning in vitro cultures of *S. suffruticosa*, the callus grown without the presence of light showed higher content of indolizidine alkaloids as compared to the illuminated cells [17]. Therefore, an experiment was conducted with shake flask-grown shoots completely darkened during the whole culture period.

As presented in Fig. 3c, d, dark-cultivated *S. suffruticosa* microshoots were thin, elongated, moderately chlorotic with signs of vitrification, and had less leaves in comparison to the illuminated culture. Nevertheless, the shoots well tolerated the lack of light, with maximum DW and Gi values ca. 9 % lower than those recorded for photoperiod-grown explants and repeated the length of L, G, and St phases of light-cultivated culture (Fig. 2b).

Similarly to the culture maintained in the presence of light, microshoots grown in complete darkness biosynthesized S and AS and stored them mostly intracellularly, with only traces present in the growth media. The patterns of alkaloid accumulation were similar to shoots maintained under photoperiod; however, the productivity per day was the highest on 40 days (Table 1). Analogically to light-cultivated shoots, significant differences between every culture phase were recorded. Moreover, significant differences were revealed for relevant phases between light- and dark-cultivated shoots, except for DW in L phase. The dark-cultivated shoots were nearly completely devoid of AS for the first two thirds of the experiment and also showed lower maximum S concentration (Fig. 2d). The results are different than those presented by Ide [17], according to which dark cultivation of *Securinega* cells did not affect S production but noticeably stimulated the accumulation of AS. On the other hand, the current data is in agreement with previous studies which indicated stimulatory effect of light on the biosynthesis of several alkaloid derivatives in plant cell cultures [18]. Given the obtained results, photoperiod cultivation of *S. suffruticosa* shoots is clearly more beneficial from a commercial point of view, providing higher amounts of pharmaceutically valuable securinine.

### The Effect of Precursor and Nutrient Supplementation on Biomass Yield and Alkaloid Content in Shake Flask Grown Microshoot Cultures

Growth medium supplementation with nutrient formulations or biosynthesis precursors is one of commonly applied strategies aimed at increasing secondary metabolite productivity in plant cell cultures [10, 18]. In the present work, *S. suffruticosa* microshoots were supplemented with LY, a distant indolizidine alkaloid precursor [2, 17], as well as the amino acid (CH) and carbohydrate (CW) compositions. Concentration of the supplements was estimated based on literature data [19, 20] and the results of preliminary experiments (data not presented).

The above supplements were added to the growth media separately or in a mixture, in order to determine potential synergistic effects between the respective substances. Basing on the previously plotted growth and accumulation profiles, it was decided to feed the culture on 34 days, i.e., at the beginning of the stationary phase and just before the rapid increase of indolizidine alkaloid accumulation. Such experimental scheme was intended to prevent the incorporation of medium supplements into primary metabolism. The biomasses were harvested on 44 days, corresponding to maximum S accumulation in the reference culture (Fig. 2).

**Fig. 4:**
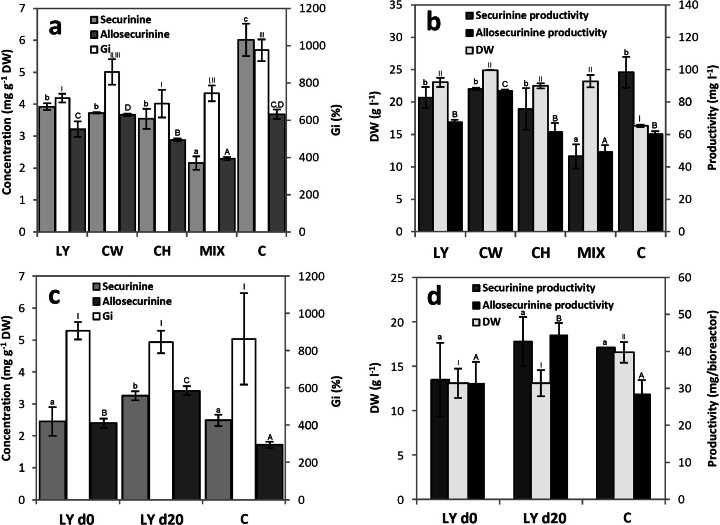
The effect of medium supplementation strategies on biomass growth (Gi, DW) and indolizidine alkaloid concentrations/productivities in *S. suffruticosa* shoot cultures maintained in shake flasks (**a**, **b**) and the bubble column bioreactor (**c**, **d**). Shake flask cultures were fed with L-lysine (LY), coconut water (CW), casein hydrolysate (CH), or their mixture (MIX) on 34 days and subsequently harvested on 44 days. Bioreactor culture was supplemented with LY on 0 (LY d0) or 20 days (LY d20) and harvested on 40 days. The results were compared to the respective controls (C). Values represent the mean ± SD of two replicates. For each parameter, distinct signs indicate significant differences among the means (*p* ≤ 0.05)

Supplementation of *S. suffruticosa* microshoots with the selected precursor and/or growth promoters did not affect negatively their morphological appearance, including biomass color and vitality. In comparison to non-supplemented cultures, the supplemented ones were characterized with lower Gi and higher DW values (Fig. 4a, b), which were indicative of their lower hydration level. The highest, over 50 % increase in DW, was recorded for CW (Fig. 4a). The observed effect is similar to previous findings and probably results from high carbohydrate content of CW [21].

Phytochemical analyses revealed that indolizidine alkaloid concentrations in supplemented microshoots were generally lowered compared to the control. The decrease, observable especially for S, was most prominent in the cultures fed with a mixture of supplements (Fig. 4a). As far as the LY supplementation is concerned, the results are in contrary to previous reports which showed increased S [22] or AS [17] accumulation in *S. suffruticosa* callus grown on LY-enriched media. The lack of positive effects with respect to secondary metabolite content, together with noticeable increase in DW indicates that the added substances were channelled into primary metabolism pathways. However, the decrease in S content in the supplemented microshoots was—especially for CW supplementation—offset by higher biomass yield, resulting in one of the highest productivities per day in the described series of experiments (Table 1). The mix of supplements failed to exert a synergistic stimulatory effect on indolizidine alkaloid biosynthesis (Fig. 4a, b).

### Biomass Yield and Alkaloid Content in Bioreactor Grown Microshoot Cultures

Scale-up experiments in bioreactors are a necessary step toward commercialization of the plant cell culture-based production system. As indicated by literature data, microshoot cultures can be successfully grown in different types of bioreactors, providing biomass for both efficient plant propagation and recovery of valuable secondary metabolites [23, 24]. For the purpose of the current work, a BCB was selected, which is one of the most common fermentor types employed for in vitro shoots cultivation [24]. Its advantages include simplicity, resistance to contamination during long growth periods, lack of mechanical agitation, and low energy consumption [23]. The BCB used in the present study was the modified version of the bioreactor described by Jaremicz and co-workers [15] which was fitted with stainless steel basket for biomass immobilization.

Contrary to the experiment run in shake flasks which ended with microshoots nearly completely filling the growth vessel, the BCB provided more space for biomass growth. Consequently, the experiment was prolonged in order to evaluate the productivity of the investigated culture in a 70-day growth period. However, initial bioreactor trials demonstrated that constant aeration, together with high surface/weight ratio of *S. suffruticosa* microshoots, caused a significant loss of medium volume, resulting in excessive shoot drying (data not shown). Therefore, it was decided to grow the shoots in a fed-batch mode, with 200 ml portions of medium added on 20 and 40 days of the experiment.

**Fig. 5:**
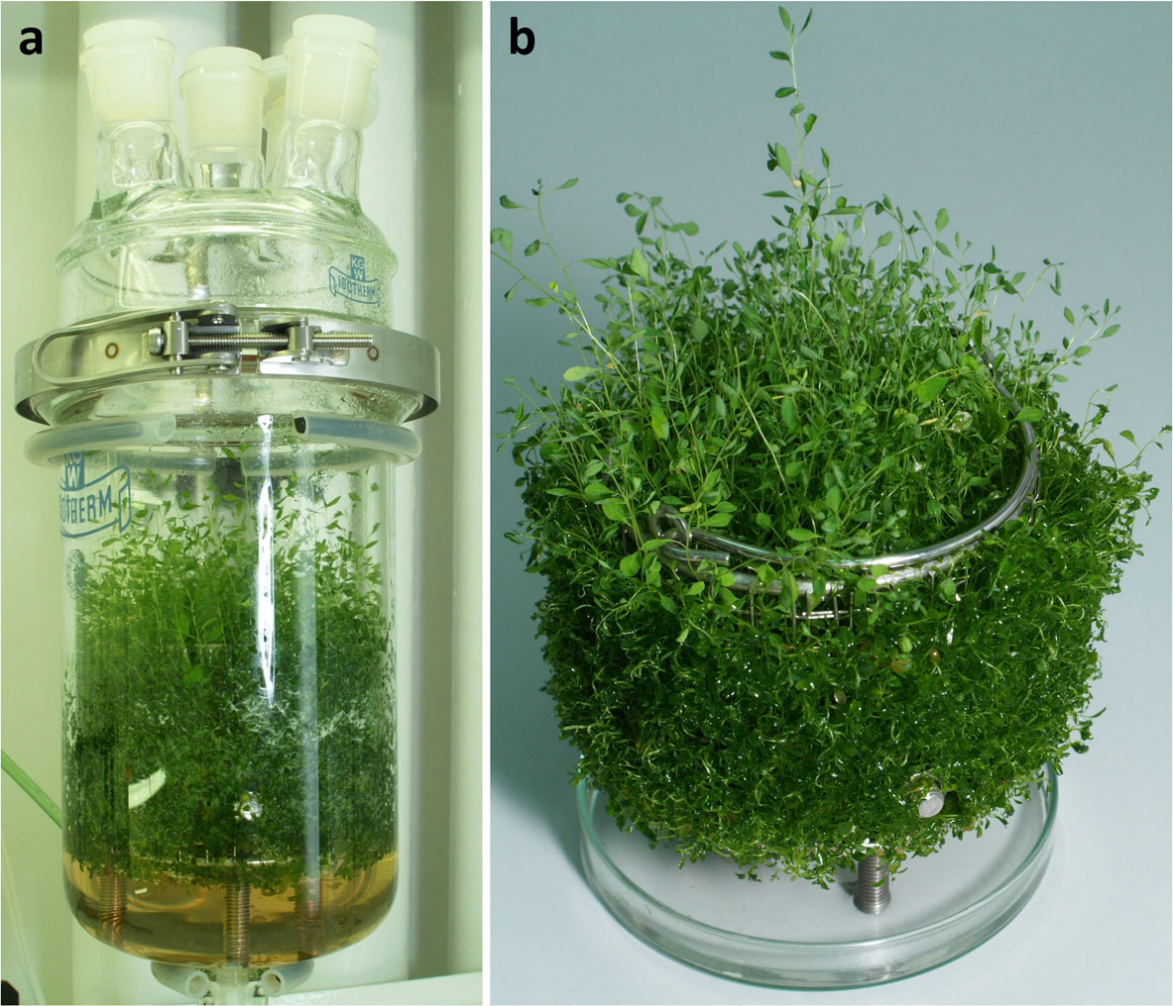
*S. suffruticosa* in vitro shoots grown in the bubble column bioreactor (BCB) for 70 days: **a** general view, **b** immobilized shoots removed from the growth vessel

**Fig. 6:**
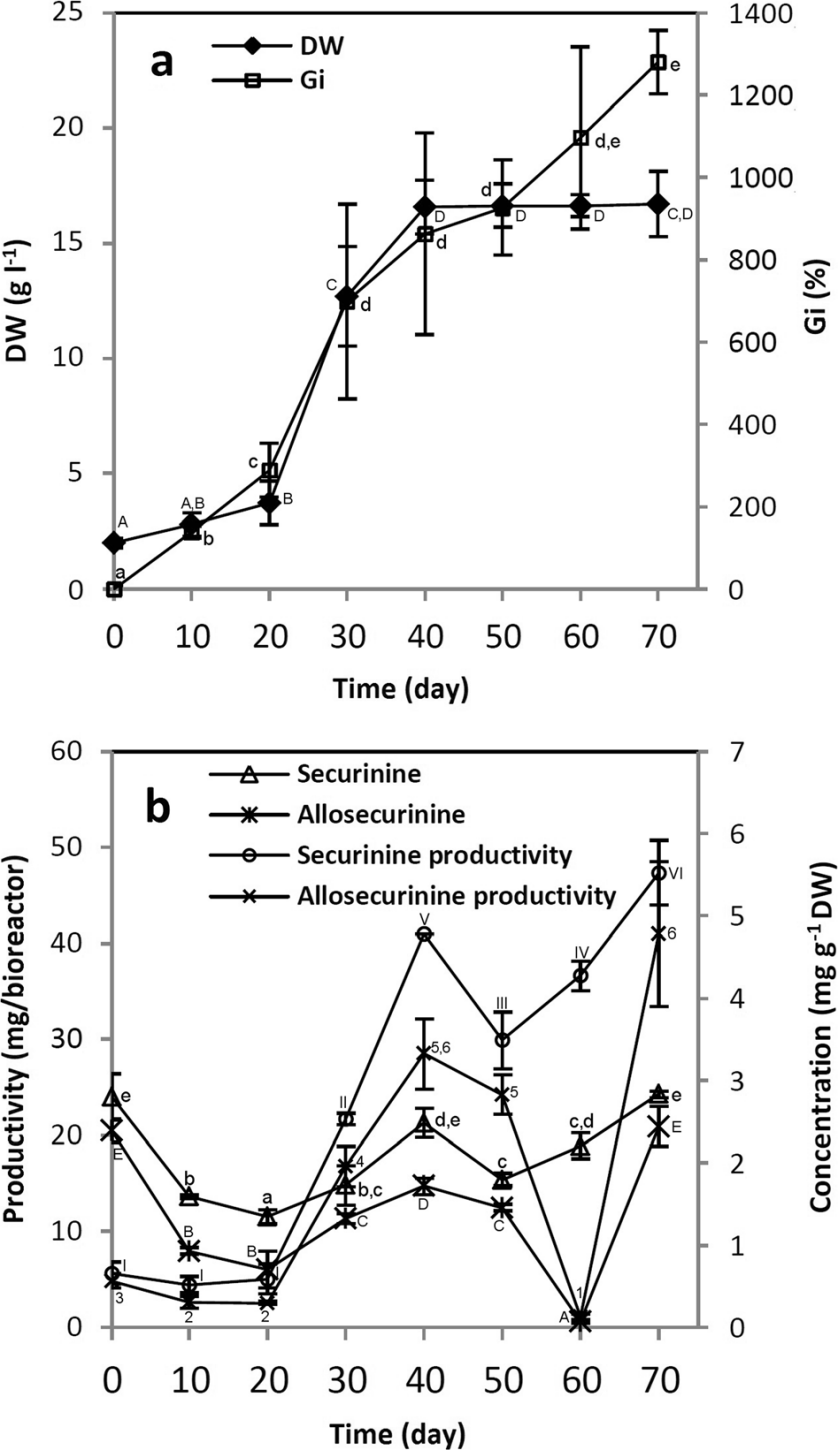
Changes in growth parameters (**a**) and indolizidine alkaloid concentrations/productivities (**b**) in *S. suffruticosa* shoot cultures maintained in the bubble column bioreactor. Values represent the mean ± SD of two replicates. For each parameter, distinct signs indicate significant differences among the means (*p* ≤ 0.05)

BCB-grown shoots remained vital during the whole growth period, showing no visible signs of necrosis (Fig. 5a, b). In comparison to SF, bioreactor culture of *S. suffruticosa* provided lower biomass yield but was characterized by more prominent stationary phase (DW curve), lasting from 40 to 70 days of the experiment (Fig. 6a).

After the 20-day lag phase, indolizidine alkaloid accumulation in BCB-grown shoots rose until 40 days of experiment. The increase in alkaloid content was recorded earlier than in SF and clearly coincided with intensive biomass growth which suggests that the competition between primary and secondary metabolism is not a factor limiting indolizidine alkaloid biosynthesis in *S. suffruticosa*. The BCB culture was also characterized by the second maximum on 70 days of experiment which was not observable in SF culture, possibly due to different cultivation modes applied and noticeably shorter growth period. Peak concentrations of S and AS in BCB-grown shoots were achieved faster than in SF experiment. However, the levels of the investigated alkaloids, as well as their respective productivities, were considerably lower (Fig. 6b). The observed differences were likely caused by altered agitation/aeration system in the BCB. Moreover, given that *S. suffruticosa* microshoots maintained in the dark were characterized by lower alkaloid levels, weak light penetration in the inner part of the BCB-grown biomass could also contribute to the above phenomenon. Nevertheless, the obtained growth system still provided nearly twice more S and AS (2.83 and 2.44 mg g^−1^ DW, respectively) than intact plant material (1.5 and 1.2 mg g^−1^ DW, respectively [16]). Like in the previous experiments, indolizidine alkaloids in BCB cultures were stored intracellularly.

In the further part of the study, bioreactor-grown *S. suffruticosa* shoots were subjected to feeding experiments aimed at increasing indolizidine alkaloid productivity. Since the BCB cultures were maintained in a fed-batch mode, it was decided that additional supplementation with nutrient formulations (CW and CH) was not necessary. Therefore, *S. suffruticosa* shoots were grown in the presence of the biosynthesis precursor (LY) which was added either at the beginning of the experiment or on 20 days of the growth period, when alkaloid levels started to rise. Based on the previously plotted alkaloid accumulation profile (Fig. 6b), the shoots were harvested on 40 days of experiment.

As depicted in Fig. 4d, LY supplementation decreased biomass yield of the bioreactor culture. On the other hand, LY stimulated the accumulation of AS when added on 0 day and increased the levels of both S and AS when supplemented on 20 days of the growth period (Fig. 4c), improving the maximal productivity per day especially in the case of S (LY d20, Table 1).

## Conclusion

In the presented work, microshoot cultures of *S. suffruticosa* were established and evaluated for the production of indolizidine alkaloids. The major outcome of the study was that the investigated biomass is suitable for the cultivation in liquid medium systems. Both shake flask- and bioreactor-grown shoots accumulated alkaloids in amounts exceeding those found in the intact plant and as such may serve as a source of therapeutically valuable securinine. Given the obtained results, *S. suffruticosa* in vitro shoots are expected to grow well in commercially available systems, such as RITA [25]. The experiments also demonstrated that at the applied concentrations of the supplements, the established in vitro culture moderately responds to nutrient and precursor feeding aimed at increasing alkaloid productivity. Therefore, it seems that clone selection at culture initiation stage, as well as metabolic engineering techniques, can probably be more effective at establishing a high-productive *S. suffruticosa* in vitro system.

## Abbreviations

2iP: 2-Isopentenyladenine
AS: Allosecurinine
BAP: 6-Benzylaminopurine
BCB: Bubble column bioreactor
CH: Casein hydrolysate
CW: Coconut water
2,4-D: 2,4-Dichlorophenoxyacetic acid
DW: Dry weight
G: Logarithmic growth phase
Gi: Growth index
IAA: Indole-3-acetic acid
IBA: Indole-3-butyric acid
L: Lag phase
LY: Lysine hydrochloride
M_A_: Murashige’s shoot multiplication ‘A’ medium
MS: Murashige and Skoog
NAA: 1-Naphthaleneacetic acid
PGR: Plant growth regulator
S: Securinine
SF: Shake flasks
SH: Schenk and Hildebrandt
St: Stationary phase

## References

[1] Yuan W, Zhu P, Cheng K, Meng C, Wu F, Zhu H (2007). Natural Product Research.

[2] Raj D, Łuczkiewicz M (2008). Fitoterapia.

[3] Zhang W, Li J, Lan P, Sun P, Wang Y, Ye W, Chen W (2011). Journal of Chinese Pharmaceutical Sciences.

[4] Rana S, Gupta K, Gomez J, Matsuyama S, Chakrabarti A, Agarwal M, Agarwal A, Agarwal M, Wald D (2010). FEBS Journal.

[5] Xia Y-H, Cheng C-R, Yao S-Y, Zhang Q, Wang Y, Ji Z-N (2011). Fitoterapia.

[6] Hong Y, Downey T, Eu K, Koh P, Cheah P (2010). Clinical and Experimental Metastasis.

[7] Gupta K, Chakrabarti A, Rana S, Ramdeo R, Roth BL, Agarwal ML, Tse W, Agarwal MK, Wald DN (2011). PLoS ONE.

[8] Holmes M, Crater A, Dhudshia B, Thadani A, Ananvoranich S (2011). Experimental Parasitology.

[9] Chen JH, Levine SR, Buergler JF, McMahon TC, Medeiros MR, Wood JL (2012). Organic Letters.

[10] Murthy HN, Lee E-J, Paek K-Y (2014). Plant Cell Tissue Organ Culture.

[11] Murashige T, Skoog F (1962). Physiologia Plantarum.

[12] Raj D, Kokotkiewicz A, Skorys A, Łuczkiewicz M (2009). Acta Biologica Cracoviensia Series Botanica.

[13] Schenk RU, Hildebrandt AC (1972). Canadian Journal of Botany.

[14] Huang L-C, Murashige T (1976). Tissue Culture Association Management.

[15] Jaremicz Z, Luczkiewicz M, Kokotkiewicz A, Krolicka A, Sowinski P (2014). Biotechnology Letters.

[16] Raj D, Kokotkiewicz A, Łuczkiewicz M (2009). Journal of Planar Chromatography - Modern TLC.

[17] Ide A, Bajaj YPS (1991). Biotechnology in Agriculture and Forestry, vol. 15. Medicinal and Aromatic Plants III.

[18] Smetanska I (2008). Advances in Biochemical Engineering/Biotechnology.

[19] George, E. F., & De Klerk, G.-J. (2007). The Components of Plant Tissue Culture Media I: Macro- and Micro-Nutrients. In E. F. George, M. A. Hall, & G.-J. De Klerk (Eds.), *Plant Propagation by Tissue Culture, The Background* (3rd ed., Vol. 1, pp. 65–113). Dordrecht: Springer.

[20] Thorpe, T., Stasolla, C., Yeung, E. C., De Klerk, G.-J., Roberts, A., George, E. F., et al. (2007). The Components of Plant Tissue Culture Media II: Organic Additions, Osmotic and pH Effects, and Support Systems. In E. F. George, M. A. Hall, & G.-J. De Klerk (Eds.), *Plant Propagation by Tissue Culture, The Background* (Vol. 1, pp. 115–173). Dordrecht: Springer.

[21] Kokotkiewicz A, Luczkiewicz M, Kowalski W, Badura A, Piekus N, Bucinski A (2013). Applied Microbiology and Biotechnology.

[22] Wysokińska, H. (1979). PhD Thesis, Medical University of Lodz, Lodz, Poland.

[23] Steingroewer J, Bley T, Georgiev V, Ivanov I, Lenk F, Marchev A, Pavlov A (2013). Engineering in Life Science.

[24] Paek KY, Chakrabarty D, Hahn EJ (2005). Plant Cell Tissue Organ Culture.

[25] Hvoslef-Eide AK, Preil W (2005). Liquid Culture Systems for in vitro Plant Propagation.

